# Network organisation and the dynamics of tubules in the endoplasmic reticulum

**DOI:** 10.1038/s41598-021-94901-2

**Published:** 2021-08-10

**Authors:** Hannah T. Perkins, Victoria J. Allan, Thomas A. Waigh

**Affiliations:** 1 Biological Physics, Department of Physics and Astronomy, Schuster Building, The University of Manchester, Oxford Road, Manchester, M13 9PL UK; 2 Division of Molecular and Cellular Function, School of Biological Sciences, Michael Smith Building, The University of Manchester, Dover Street, Manchester, M13 9PT UK

**Keywords:** Biological physics, Biophysics, Biopolymers in vivo, Membrane biophysics, Membrane structure and assembly

## Abstract

The endoplasmic reticulum (ER) is a eukaryotic subcellular organelle composed of tubules and sheet-like areas of membrane connected at junctions. The tubule network is highly dynamic and undergoes rapid and continual rearrangement. There are currently few tools to evaluate network organisation and dynamics. We quantified ER network organisation in Vero and MRC5 cells, and developed an analysis workflow for dynamics of established tubules in live cells. The persistence length, tubule length, junction coordination number and angles of the network were quantified. Hallmarks of imbalances in ER tension, indications of interactions with microtubules and other subcellular organelles, and active dynamics were observed. Clear differences in dynamic behaviour were observed for established tubules at different positions within the cell using itemset mining. We found that tubules with activity-driven fluctuations were more likely to be located away from the cell periphery and a population of peripheral tubules with no signs of active motion was found.

## Introduction

The endoplasmic reticulum (ER) is a complex membranous network of tubules and sheet-like regions that is continuous with the nuclear envelope and stretches out to the cell periphery. Narrow tubules ($\sim 100 \,{\rm{nm}}$ in diameter^1,2^) intersect at branch points, which typically connect three tubules^3^. The ER is responsible for the biosynthesis of many proteins and lipids, as well as calcium ion storage and regulation^4–6^. Links between network morphology and the functions of the ER have been found (reviewed in Westrate et al.^7^). For example, sheet-like regions tend to have a higher ribosome density^8^ and be enriched in proteins involved in translocation^9^, thus they are thought to be the main regions for protein synthesis. Tubules may be the main regions for lipid synthesis^10^ and Ca^2+^ regulation^11^, although these links are not fully understood.

Almost all other subcellular organelles interact with the ER at membrane contact sites (MCSs)^12^, including mitochondria^13–16^, endosomes^17–20^, the Golgi apparatus^21^ and the plasma membrane^22^. Microtubules also interact with the ER via the motor proteins cytoplasmic dynein^23–26^ and kinesin-1^26,27^ and via tip attachment complexes (TACs). TACs can be formed between the ER and microtubules in the absence of motor proteins^28^. EB1, a protein localised to the fast-growing microtubule plus-ends, binds to STIM1, a protein localised to the ER, to form a TAC^29^. This causes a rearrangement of the ER network as the microtubule polymerises. MCSs with mitochondria, late endosomes, lysosomes and interactions with microtubules were recently shown to facilitate remodelling of the ER network^30^.

The tubules and junctions of the ER network are highly dynamic and oscillate over time^2,31^. The functionality of these oscillations is not yet known, although it has been suggested that the motion facilitates mixing of the reactants in a *shaken reaction vessel model*^2^, increasing the rate of reactions and therefore the rate of protein synthesis. The dynamics do however seem to vary between cell types, with a higher ER motility observed in VERO cells than in COS7 cells^26^. Peristalsis of the ER tubules has also been postulated^32^, although little quantitative data has yet been presented for this model. Detailed links between the dynamics and the function of the ER have yet to be made.

From the perspective of active matter physics^33,34^, the ER represents an important, but relatively unexplored example of active matter. Motor protein activity drives the system out of equilibrium. The tubules are self-assembled from surface active molecules (predominantly lipids, although proteins play an important role in branching and curvature^35–37^), but resemble polymers at large length scales. The tubules branch and form a complex subcellular network, which is rearranged actively by molecular motors.

A challenge when trying to link dynamics with function is the difficulty in tracking ER tubules in vivo. In this work, we present methods to quantify the structure and dynamics of established ER tubules (defined as those that remain connected to the network at both ends for the duration of the analysis) from live cell videos. In total, we quantified the network structure, translational motion of the tubules, internal fluctuations in the curvature and detected signatures of driven tubule oscillations. A machine learning method, itemset mining, was used to discover patterns in the results and links were found between activity and cellular location. Based on this analysis, we find that established ER tubules generally oscillate due to thermal motion, although activity-driven events cause significant distortions in a subpopulation of tubule contours.

## Results

### Network organisation

**Figure 1:**
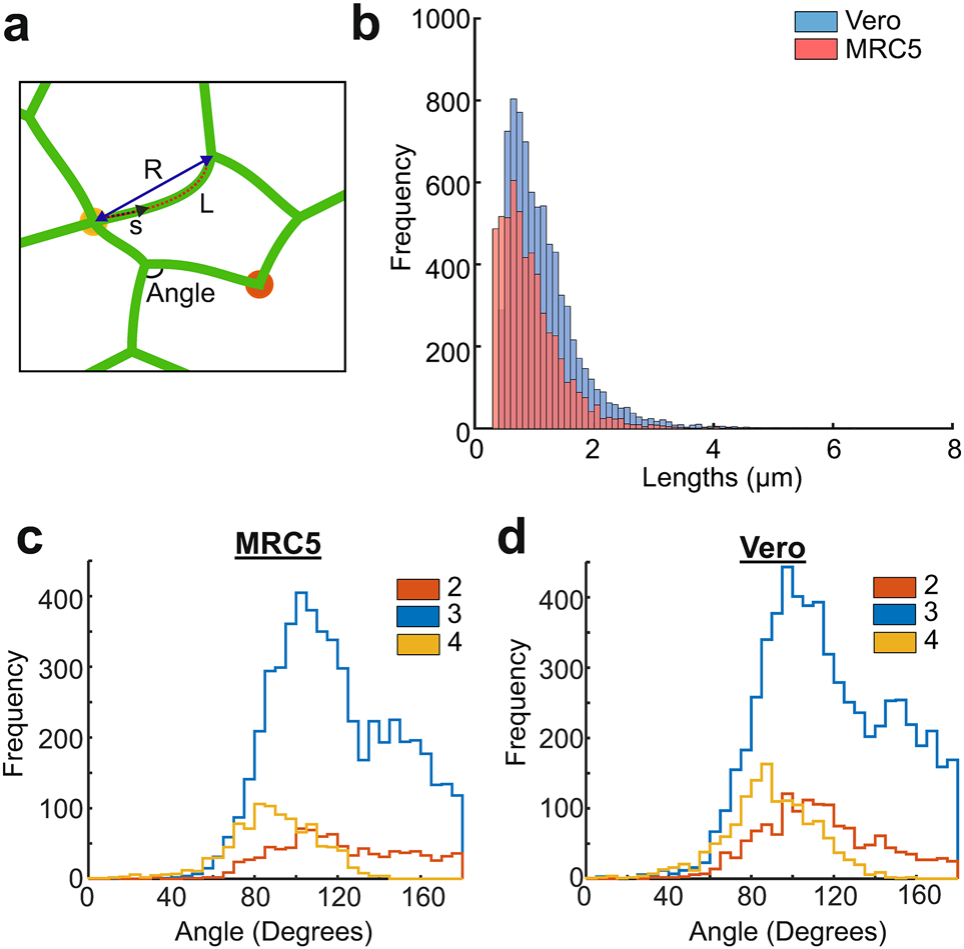
Quantification of lengths and angles in the ER network. (**a**) Schematic with definitions of lengths and angles used in this analysis. Tubule contour length, $L$, is shown as a red dashed line, the arclength, $s$, is shown as a black dashed arrow and the end-to-end distance, $R$, is shown as a dark blue solid arrow. Junctions between two and four tubules are highlighted with orange and yellow circles respectively. (**b**) Histogram of Vero (blue) and MRC5 (red) ER tubule lengths, measured in fixed Vero cells and stills of live MRC5 cells. (**c**) Angles measured in the ER network of MRC5 cells, separated by the junction co-ordination number (shown in key). (**d**) Angles within the ER network of Vero cells, separated by junction co-ordination number (shown in key).

ER network morphology can vary dramatically between cell types and is known to depend on the functional requirements of the cell^7^. SOAX, software designed to identify fibres and junctions in still images of filamentous networks^38^, was used to extract the position of tubules and branch points in still images of the ER (see “Methods”). Tubule contour lengths (defined as the length between network branch points along the tubule, shown by $L$ in Fig. 1a) and the angles between tubules at branch points (Fig. 1a) were measured, to quantify the morphological properties of the ER network.

Tubule contour lengths in the ER networks of fixed Vero and live MRC5 cells were found to be of the order of micrometres, with a mean length of $0.940\pm 0.007$ μm for live MRC5 cells (n = 5448 tubules, mean ± SEM) and $1.148\pm 0.007$ μm for fixed Vero cells (n = 7745 tubules, mean ± SEM). Figure 1b shows a histogram of tubule lengths. When the contour lengths of tubules were studied in live cell videos for both MRC5s and Veros, lengths were found to be fairly constant over short time scales (10 s), with the exception of a small number of rapid extension events.

The average angles subtended by tubules at branch points were found to vary with the number of tubules connected in both cell types (Fig. 1c,d). Combining the results from both cell types, the ER network was found to have junctions consisting of 2, 3 or 4 tubules (Fig. 1a), with 3-way junctions being the largest population ($56\pm 2 \%$), followed by 2 ($35\pm 2 \%$) and then 4 ($9\pm 2 \%$). For junctions between four tubules, the mean angle measured between tubules was $89.2\pm 0.5$°. This might correspond to a quasi-equilibrium configuration of the branch point, although it is worth noting that with this method of analysis, it is impossible to discern whether the membrane of these tubules has fused to form a branch point or if the tubules are simply overlapping at different depths. It is likely, given the high frequency of $90$° angles observed, that tubules are in fact crossing at different depths of focus. A bimodal angular distribution for 3-way branch points (more pronounced in MRC5 cells, blue in Fig. 1c) was observed with $103^\circ \pm 1^\circ$ observed roughly twice as frequently as $156^\circ \pm 2^\circ$. Under symmetric equilibrium conditions, a separation of $120^\circ$ is expected, with equal tension in all three tubules. However, branch points could for instance each have two $100^\circ$ angles and one $160^\circ$ angle if there are heterogeneous non-equilibrium forces, and therefore tensions, within the network. As the ER is highly dynamic and undergoes constant rearrangements, it seems likely that the majority of tubules experience such non-equilibrium conditions. The angles observed at 2-tubule branch points ranged from $\approx 20^\circ$ to less than $180^\circ$. These 2-way junctions have been classified as puncta in plant cells^39^, which connect the ER to the plasma membrane^40,41^. Given that a branch point between two non-colinear flexible fibres cannot be formed without a third component to balance the forces, this result indicates that the ER forms MCSs with other cellular structures or interacts with microtubules at these branch points.

Fluorescence images were used to calculate the persistence length of ER tubules in live Vero cells, using a similar method to Georgiades et al.^2^. The persistence length gives a measure of the flexibility of fibre-like objects. It is defined as the characteristic distance along the contour of the fibre over which angular correlations in the tangential direction decorrelate. Semi-flexible fibres are those with comparable lengths and persistence lengths.

FiberApp^42^ was used to trace the contours of ER tubules in images of live Vero cells (see “Methods”). Tubules were defined as the sections of the network between two branch points and the end-to-end distance was defined as the straight-line distance between the tubule ends (Fig. 1a). The contour lengths, $L$, and end-to-end distances, $R$, were determined. The mean square end-to-end distance of a fibre, $\langle {R}^{2}\rangle$, can be related to the contour and persistence length, ${L}_{p}$, as^43^

1 $$\langle {R^{2}} \rangle = {2L_{p}} \left( {L - {L_{p}} \left( {1 - e^{{ - L/{L_{p}} }} } \right)} \right).$$

A persistence length of $8.3\pm 0.2$ μm (mean ± SD) was determined for ER tubules in live Vero cells using Eq. 1. This value is in reasonable agreement with the persistence length of $4.71\pm 0.14$ μm previously found for fixed and live MRC5 cells^2^ and indicates that ER tubules can be modelled as semi-flexible filaments. In all, ER tubules were found to behave as semiflexible fibres in vivo and angular analysis showed signatures of uneven tensions at junctions and of MCSs between the ER and other organelles.

## Point tracking

In order to draw connections between ER motion and why this might be beneficial to cell function, methods for quantifying ER dynamics must be developed. These methods should result in properties that are easy to compare between cell types and conditions. Previously, ER tubule extension speeds^23,26^, transverse motion^2^ and junction motion^44^ have been quantified and recently some theoretical models for dynamic networks of semiflexible fibres have arisen^45,46^. The network has also been well-modelled using energy minimisation algorithms^47,48^ although the assumptions may not be appropriate for the driven, non-equilibrium network of the ER. In this work, we address the dynamics of individual tubules between branch points. Specifically, we asked if tubule motion was due to thermal oscillations or was it activity-driven (for example, by motor proteins)? It is known that the ER is rearranged by both kinesin-1^26^ and cytoplasmic dynein^23,26^, as well as through MCSs with many organelles^12^, and is therefore activity-driven. However, this rearrangement refers to new tubules being drawn out and connected to the network. Our focus in this work is whether or not established tubules, that remain connected to the network for the duration of the observation, fluctuate due to activity.

**Figure 2:**
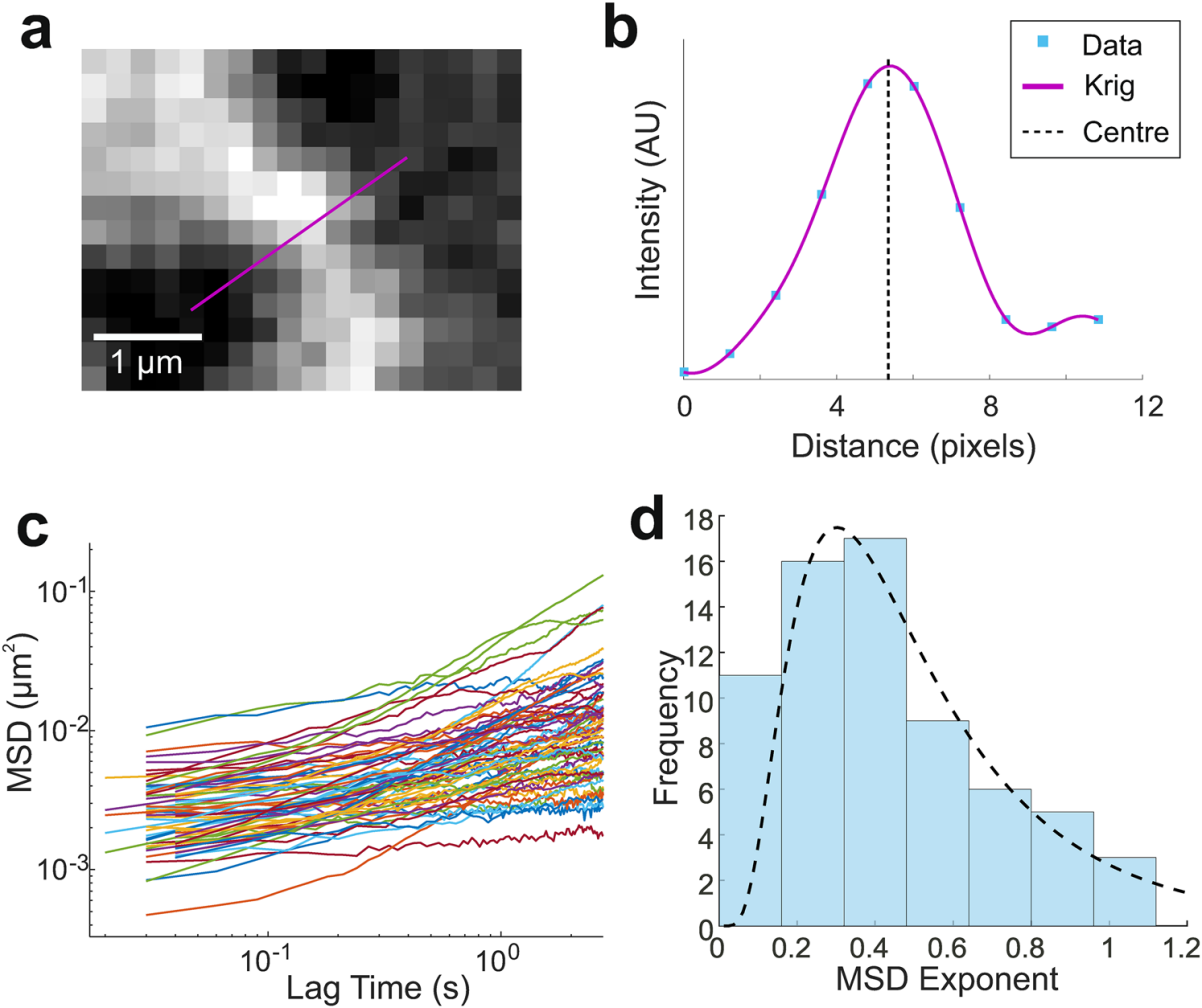
Mean squared displacement of ER tubule oscillations in live Vero cells. (**a**) Fluorescence image of a single ER tubule. The tubule position along the purple line was tracked. (**b**) Fluorescent intensity along the line in (**a**), with krig interpolation and the centre of the fit depicted. (**c**) MSDs of measured transverse tubule fluctuations as a function of lag time. Traces begin at different time points due to slight differences in video frame rates. (**d**) Exponents of the power law fit to the MSDs in (**c**). Dotted line shows lognormal fit with mean exponent 0.54 ± 0.04.

We initially studied ER tubule dynamics using mean squared displacements (MSDs), a standard method for quantifying particle dynamics^49^. The MSD in two dimensions is defined as

2 $$\langle {\Delta r^{2}(\tau)}\rangle ={\langle {\left(x\left(t+\tau \right)-x(t)\right)}^{2}+{\left(y\left(t+\tau \right)-y(t)\right)}^{2}\rangle }_{t},$$

where $x(t)$ and $y(t)$ are the co-ordinates at time $t$ and $\tau$ is the lag time. MSDs of lateral tubule movements were calculated by tracking the position of a point on a tubule over time. A line was drawn perpendicularly to the tubule (Fig. 2a) and the co-ordinates of the tubule on this line were tracked, similarly to our previously described method^2^, but with the inclusion of several extra steps to minimise tracking errors (Fig. 2b, see “Methods”). The MSD can be assumed to describe the transverse motion of the tubule, $\langle {\Delta r^{2}(\tau)}\rangle = \langle {{\Delta r}_{\perp }^{2}(\tau)}\rangle +\langle {{\Delta r}_{||}^{2}(\tau)}\rangle \approx \langle {{\Delta r}_{\perp }^{2}(\tau)}\rangle$, as longitudinal tubule motion generally has a much smaller amplitude than transverse motion^33^. A power law can be used to approximate the transverse MSD: $\langle {{\Delta r}_{\perp }^{2}(\tau)}\rangle \propto {\tau }^{\alpha }$, in which the exponent, $\alpha$, describes the motion of the tracked object. Brownian motion is described by $\alpha \approx 1$, whereas *super-diffusive* motion has an exponent $1<\alpha <2$, and $0<\alpha <1$ describes *sub-diffusive* motion. For semiflexible fibres oscillating thermally in a viscous medium with no applied tension, $\langle {{\Delta r}_{\perp }^{2}(\tau)}\rangle \propto {\tau }^{3/4}$ is expected, whereas for tension-dominated regimes theory predicts that $\langle {{\Delta r}_{\perp }^{2}(\tau)}\rangle \propto {\tau }^{1/2}$ (^50^).

The majority of Vero ER tubules moved sub-diffusively, with $\alpha <1$ (Fig. 2c,d). A lognormal distribution was fitted to the histogram of MSD exponents with $\overline{\alpha }=0.54 \pm 0.04$ (mean ± SEM), indicating that single points on ER tubules generally behave as thermalized fibres oscillating under tension in live cells^33^. A small proportion of tubules however displayed super-diffusive motion associated with active, motor-driven motility. These results are in agreement with our previous work^2^.

### Contour tracking

To study tubule dynamics in more detail, tubule contour tracking software was developed. Previously, the changes in contour shapes of isolated semiflexible fibres in vitro have been studied, including actin and microtubules^51,52^. However, many of the previous theoretical predictions assume conditions that are not suitable to describe ER tubules in vivo*,* namely free end boundary conditions, thermal motion and no applied tension. However, analysing the changes in shape of a whole ER tubule does allow for a more rigorous description of the thermal or active dynamics along the whole fibre, as well as a method to examine the tubule curvature changes. We have developed a tracking and analysis workflow to analyse in vivo ER tubule contour dynamics.

**Figure 3:**
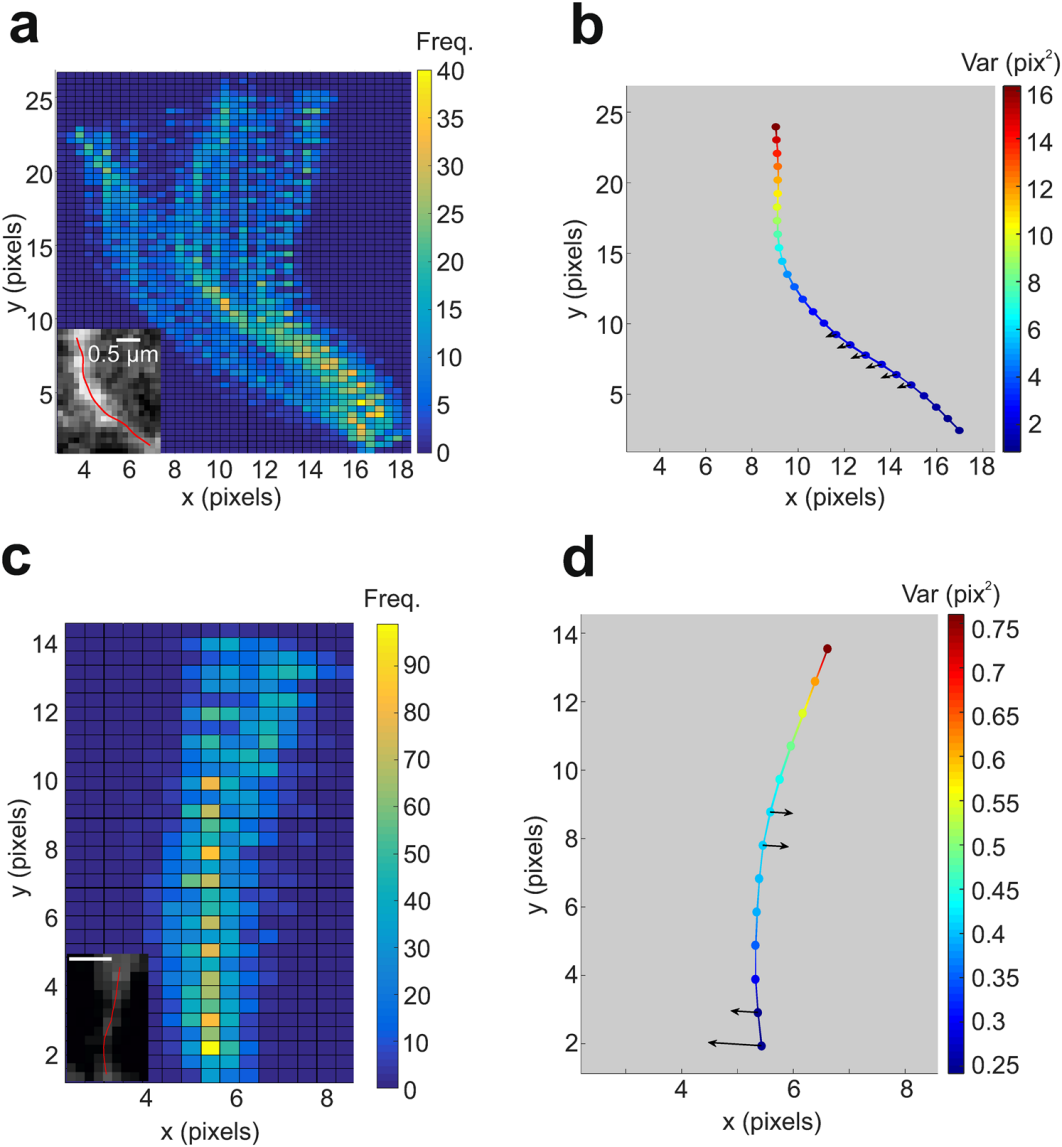
Tracked contours of ER tubules show indications of active motion. (**a**,**c**) Contours are overlaid to show the time-dependence of tubule shape (inset shows fluorescence image of tracked tubule). More frequently occupied positions are coloured in yellow. (**b**,**d**) Mean position of tubules in (**a**) and (**c**) respectively, coloured according to the variance of transverse oscillations. Arrows show direction and magnitude of significant transverse skewness, if detected.

Contours were automatically traced for each frame of a video, using a version of FiberApp^42^ modified for this work (see “Methods”). Examples of tubules and their contours are shown as insets in Fig. 3a,c. The co-ordinates of the tubule at the $k$th point along the backbone of the tubule at time $t$, $\left({x}_{k }(t),{y}_{k}(t)\right)$, were used to find the average tubule contour, or backbone, $\left({\overline{x} },\overline{y }\right)$. For each position along the backbone, $k$, the variance and skewness of the transverse displacement from the backbone were found (Fig. 3b,d). The variance of each position, along with the map of tubule contours (Fig. 3a,c) provides information about the types of tubule oscillations. For example, the map of tubule contours in Fig. 3a shows a tubule that is hinged i.e. much more dynamic at one end than the other. In contrast, Fig. 3c shows a tubule transitioning from a slightly curved to a straight conformation. Further analysis into tubule conformational changes, especially when analysed in conjunction with other organelle dynamics, may provide insight into the causes and functional benefits of ER oscillations.

Thermal fluctuations are defined as random deviations about an average position. The probability of a thermally fluctuating object occupying a position at a given displacement from the average position is Gaussian^53^ for harmonic systems, but will at least be symmetrical about the mean position for more complex anharmonic systems. Therefore, if ER tubules oscillate purely due to thermal fluctuations, we expect a symmetric distribution of displacements from the backbone. To test this, the skewness of the perpendicular displacements from the backbone were found for each tracked position on the tubules. Skewness significance was determined by comparing the measured value to the standard error of skewness (see “Methods”). Significant skewness was detected for at least one position for most tubules (≈78%), with 243 of the 729 points analysed displaying significant skewness. The line density of points with significantly skewed displacements was found to be $3.44\pm 0.07$ μm^−1^. These results, along with the results from the transverse MSD analysis indicate that tubules generally oscillate thermally, with frequent activity-driven events occurring along the backbone.

### Fourier decomposition

**Figure 4:**
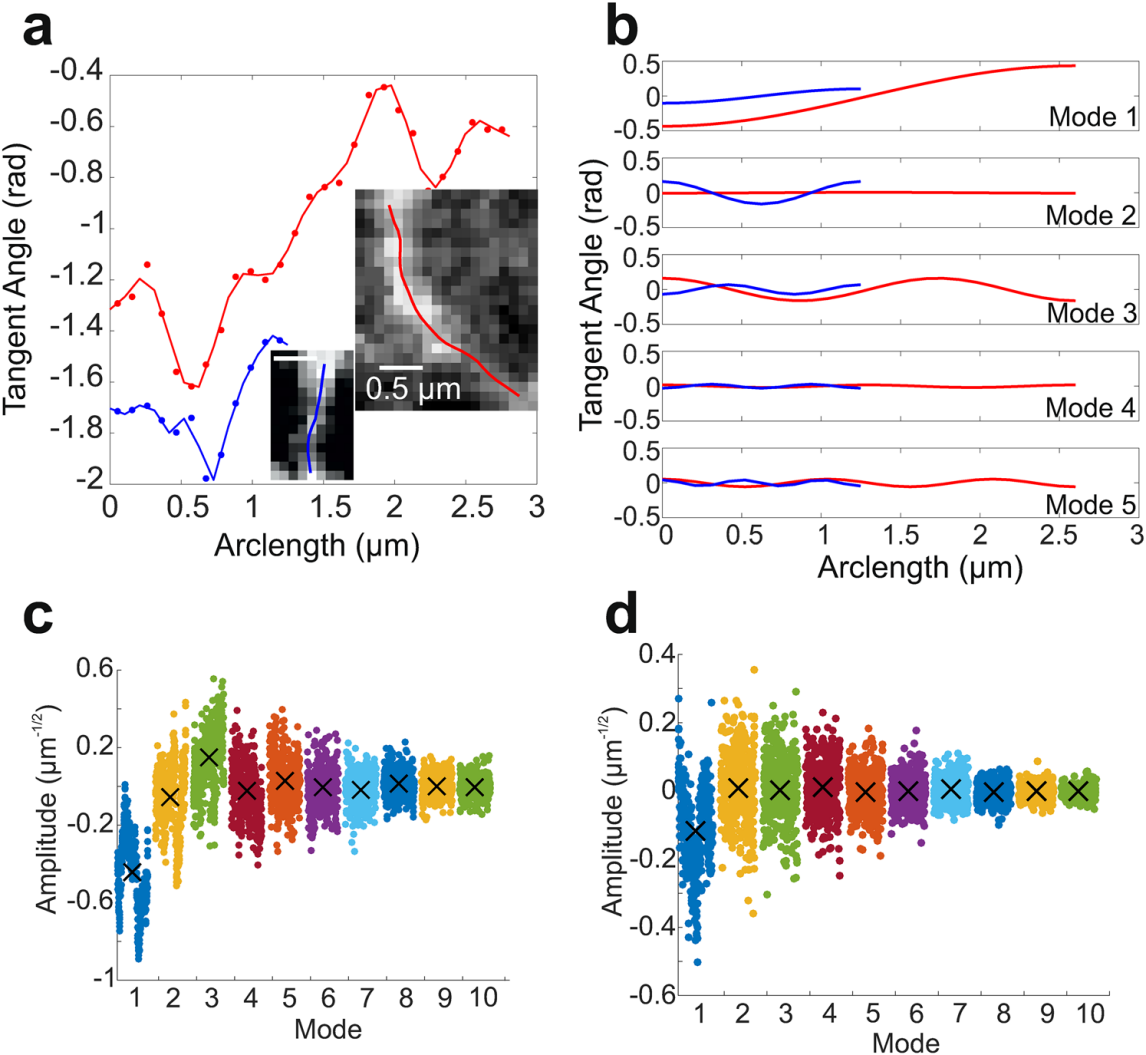
Tubule contours modelled with Fourier modes. (**a**) Tangent angles as a function of arclength, calculated directly from the tracked points (solid line) and from the modelled Fourier modes (points) for the tubules in the insets. (**b**) First 5 Fourier modes used to model the tubules in the single frame depicted in (**a**). (**c**) Mode amplitudes of the red tubule in (**a**) for each tracked frame. (**c**) Mode amplitudes of the blue tubule in (**a**) for each tracked frame. Black crosses show mean mode amplitudes.

To further investigate ER dynamics at the tubule level, the dynamic nature of tubule curvature was analysed using Fourier modes. The method of Fourier decomposition for the shape changes of filaments has been described previously^51,52^ (see “Methods” for a detailed discussion of the approach used in this work). Briefly, Fourier mode decomposition describes the following steps: the angle between points along the backbone, the tangent angle, is found (Fig. 4a); the tangent angle is modelled as a sum of sinusoidal waves (modes), each with an individual amplitude (Fig. 4b). Higher modes describe higher frequency oscillations in the tangent angle. The amplitude of each mode determines the relative amount of the particular mode required to accurately represent the contour. A mode amplitude of zero indicates that there are no fluctuations of the tangent angle at the mode frequency. A straight fibre would therefore have mode amplitudes of roughly 0 for all modes, at all times. Tubules were classified into 2 groups using the Fourier mode amplitudes: those with sustained curvature and those with a predominantly straight backbone. ER tubules for which mode amplitude values fell on one side of zero for more than 87% of frames, for at least one Fourier mode, were said to have sustained curvature (Fig. 4c, see “Methods”). This threshold was arbitrarily chosen, but ensured that for a tubule to be classified as having sustained curvature, curvature was detected in almost all frames for at least one Fourier mode. If this condition was not met in any of the modes, no sustained curvature was found (Fig. 4d). In all, few tubules (≈18%) had sustained curvature, although many tubules with no sustained curvature did exhibit transient curvature, as seen in the first mode in Fig. 4d. Long-lived contact sites between the ER and other cellular structures may facilitate continuously curved tubule configurations, also known as states of prestress^54,55^. From this analysis, we conclude that ER tubules in vivo predominantly oscillate about a straight conformation.

### Itemset mining

In this work, we have introduced methods to track and analyse the motion of individual, established ER tubules, which will be useful for future work comparing ER dynamics for different cell types and experimental conditions. However, the results can also be used to check for sub-populations of tubules within a single cell type. Frequent itemset mining is a machine learning technique that searches for associations between variables within large datasets^56^. This method was used as opposed to a mixture model because some of the variables have a finite support (for example, the MSD exponent, $\alpha$, is only defined between 0 and 2) or are qualitative and are therefore not appropriate for continuous variable methods.

The presence, or lack of, four properties were analysed for each tubule: sustained curvature, peripheral position in the cell, an MSD exponent of 0.4 or greater and significant skewness (as an indicator of driven motion). Tubules were defined as peripheral if they resided in the outermost 10% of the distance between the nuclear envelope and the cell edge. In total, 12 association rules were found although these can be grouped into 4 relationships, based on the similarities in the rule variables (see Table 1). Firstly, central tubules with strongly sub-diffusive MSD exponents ($\alpha <0.4$) and significant skewness often had no sustained curvature. From this, we conclude that even when the activity at a single point on the backbone is strongly sub-diffusive and the backbone has a straight equilibrium position, hallmarks of driven motion are often seen when observing the entirety of established perinuclear tubules. Secondly, tubules with significant skewness and higher MSD exponents ($\alpha >0.4$) were likely to be central. Interestingly, this rule states that actively driven tubules are most often located away from the cell periphery. Thirdly, peripheral tubules with either low MSD exponents or no significant skewness had a straight equilibrium position. This agrees with our observations in live cell experiments; straight tubules with minimal transverse fluctuations are often seen at the thinner cell periphery. Finally, tubules with sustained curvature have significant skewness. It should be noted that only rules with a confidence of 0.9 or greater were included in this work. This meant that rules were only included if the consequent applied to 90% or more of the cases in which the antecedent was true. Therefore, although the inverse of several of the rules stated in Table 1 apply at a lower confidence threshold, they are not included in these results. For example, peripheral tubules are more likely to have strongly sub-diffusive MSD exponents, however this rule has a confidence of 0.79 and therefore was not included in these results. Together, these results show that the motion of established tubules can be described by sub-populations, and that tubule dynamics are directly related to cellular location.

**Table 1:** Association rules found using itemset mining. Support, confidence and lift are given to 2 significant figures.

Group	Antecedent	Consequent	Support	Confidence	Lift
1	Significant skewness & strongly sub-diffusive MSD exponent	No sustained curvature	0.41	0.93	1.1
	Perinuclear & strongly sub-diffusive MSD exponent	No sustained curvature	0.24	1.0	1.1
	Significant skewness & perinuclear & strongly sub-diffusive MSD exponent	No sustained curvature	0.18	1.0	1.1
2	Thermalised or active MSD exponent & significant skewness	Perinuclear	0.32	0.92	1.6
	Thermalised or active MSD exponent & perinuclear	Significant skewness	0.32	0.92	1.2
3	Peripheral & strongly sub-diffusive MSD exponent	No sustained curvature	0.29	0.91	1.0
	No significant skewness	No sustained curvature	0.21	1.0	1.1
	Peripheral & no significant skewness	No sustained curvature	0.12	1.0	1.1
4	Sustained curvature	Significant skewness	0.12	1.0	1.3
1 & 3	Strongly sub-diffusive MSD exponent	No sustained curvature	0.53	0.95	1.1

Support gives the proportion of tubules in which a given itemset occurs and confidence is a measure of how often a rule applies within the population of tubules for which the antecedent is true. Lift is a measure of how much more likely the consequent is to occur along with the antecedent than on its own, considering all tubules (see “Methods”). Rules which are related are grouped together. The last row relates to both groups 1 and 3.

## Discussion

The endoplasmic reticulum is an underexplored real-world example of active matter. Many processes essential to cell survival are performed by the ER, the efficacy of which may depend on ER organisation and dynamics. Abnormal ER morphology is linked to diseases such as hereditary spastic paraplegias^57,58^, and it is possible that the dynamics are also implicated. Therefore, analysing the ER network in normal cells is important for the understanding of disease-related alterations. In this work, we outline thorough quantification methods for determining ER organisation and the dynamics of established tubules i.e. those that remained connected to the network at both ends throughout the analysis. Rapid, active tubule extensions also occur throughout Vero cells^26^, however these were not the focus of this work. The fluorescence microscopy techniques used in this work are not applicable to the matrices and sheets of the network, however correlative techniques such as fluorescence correlation spectroscopy (FCS) and differential dynamic microscopy (DDM) provide hope for the future analysis of these structurally complex regions. Notably, our quantitative analysis revealed that established ER tubules (with junction points at each end) could also undergo active dynamics with indications of tension imbalances and membrane contact sites.

The ER is a fluid system which can form new tubules when subject to external forces and therefore differs from traditional biopolymer networks. However, on the level of single tubules, the ER can be modelled as a network of semiflexible polymers with a mean contour length $1.148\pm 0.007 \mu {\rm{m}}$ and persistence length $8.3\pm 0.2 \mu {\rm{m}}$ in Vero cells. The majority of junctions connect 3 tubules, although their angular distributions indicate that tubules experience uneven applied forces. Mechanical stresses in the tubules have multiple signatures in our experiments, including asymmetrical angles at junctions, MSDs with exponents less than ¾ (^50^) and non-zero curvatures (states of prestress^54,55^).

Structural links between the ER and other subcellular organelles or cytoskeletal structures, such as microtubules, may account for 2-way junctions in the network. A possible mediator of these 2-way junction is CLIMP-63, a transmembrane protein, which has been shown to form static links between the ER and microtubules^59,60^. CLIMP-63 is resident in the ER both in sheet-like regions^9^ and at the cell periphery^31,61^, and has a cytoplasmic tail that facilitates binding of the ER directly to microtubules. REEP-1, a protein that localises to tubular ER has also been shown to mediate microtubule-ER interactions^57^. Overexpression of REEP-1 caused ER tubules to align with microtubules, indicating that it acts as a linking protein between the two organelles and therefore may help to stabilise 2-way junctions in the ER network. These connections may also play a role in the positioning of the ER within the cell.

Careful analysis of mean squared displacements and contour shape changes of tubules indicate that the motion of established tubules in the ER network is predominantly sub-diffusive. However, many active events were observed by means of super-diffusive exponents and significant skewness in tubule displacements. Fourier mode decomposition of the tubules revealed states of sustained curvature, or prestress, in a few tubules, which are also indicative of membrane contact sites or interactions with microtubules. We conclude that established tubules oscillate both passively due to the tension under which they are held and the motion of the surrounding cytoplasm, and actively at points along the tubule backbone due to membrane contact sites and motor-driven events.

Clear links were made between the position of established ER tubules within the cell and tubule activity using itemset mining. From these associations, we can begin to explain the different types of motion detected. Peripheral, predominantly straight tubules with no indications of active motion may play a role in regulating network tension and localisation via contact sites with microtubules, the plasma membrane and possibly with actin. ER tubules have been found to align almost completely with microtubules at the periphery of *Xenopus* tissue culture cells^62^ and in lamellae^3,63^, which may explain the lack of transverse driven tubule motion. An interaction between ER tubules and actin has also been hypothesized^64^, although the mechanisms of this interaction have yet to be found. ER-PM MCSs that are stable for tens of seconds have been observed^65^, and could also play a role in peripheral tubule stability. The geometry and organisation of the cell periphery is also likely to contribute to the stability of these tubules. Peripheral regions of the cell are thin and a dense mesh of actin is present at the periphery, both of which are likely to constrain tubule motion.

Tubules with signatures of activity-driven fluctuations are more likely to be perinuclear than peripheral. Further from the cell periphery, established tubules are less constrained by the geometry of the cell and therefore active movement is less restricted. Another possible consideration is that more motor proteins may drive tubule motion or that more MCSs may be formed in these central regions. An explanation for this could be the increased density of microtubules and of organelles further from the cell periphery^21^. A greater organelle density may lead to more MCSs, while a greater density of microtubules may lead to more motor-driven events, both of which would result in more activity-driven ER tubules.

We hypothesize that membrane contact sites and interactions with motor proteins may be the cause of active, transverse oscillations in established ER tubules. Transient interactions with trafficking organelles or motor proteins may be the cause of activity in predominantly straight tubules residing further from the periphery. However, tubules with sustained curvature as well as activity may be brought about by longer-lived MCSs with larger, trafficking organelles, such as endosomes and mitochondria. Alongside trafficking and remodelling ER morphology^30^, these organelles may have a further purpose—to promote ER dynamics, enhancing the mixing of reactants and therefore allowing the ER to function more efficiently.

Together, the analytical tools outlined in this work provide a new detailed, quantitative description of the endoplasmic reticulum in live cells. In the future it is hoped that tubule behaviour can be explored in a wider range of cell types and that a rigorous link between ER dynamics and function can be made.

## Methods

### Cell culture and imaging

VERO cells, an immortalised cell line derived from the kidney of an African green monkey, were obtained from the European Collection of Authenticated Cell Cultures (ECACC) and cultured in Dulbecco's Minimum Essential Media (DMEM) supplemented with 10% Fetal Bovine Serum (FBS) at 37 °C and 8% CO_2_.

### Fixed imaging

48 h prior to imaging, Vero cells were cultured on No 1.5 glass coverslips in a 12-well plate. 24 h prior to imaging, cells were transiently transfected with a construct encoding signal sequence-streptavidin-KDEL (ER-retention sequence), an ER hook from the RUSH system^66^. Str-KDEL_neomycin was a gift from Franck Perez (Addgene plasmid # 65306; http://n2t.net/addgene:65306; RRID:Addgene_65306). This construct allowed subsequent labelling of well-fixed ER using fluorescent biotin, a method which is less susceptible to artefacts than antibody labelling. SS-Str-KDEL DNA (800 ng) was combined with 2 μL of JetPEI (Polyplus transfection) in a total of 100 μL of 150 mM NaCl per coverslip. The solution was left to incubate at room temperature for 15 min before being added to the wells.

The next day, the cells were fixed in 3% formaldehyde with 0.1% glutaraldehyde solution, in PBS for 20–25 min at room temperature. Coverslips were washed three times with PBS before permeabilisation with 0.5% (v/v) Triton X100 in PBS for 10 min at room temperature. Unreacted aldehyde groups were then quenched using 1 mg/ml of sodium borohydride in PBS (three five-minute incubations). ATTO 565-biotin (Sigma 92637) was dissolved in DMSO to a concentration of 0.4 mM. After washing in PBS, coverslips were incubated with a 1/2000 ATTO 565-biotin dilution for 25 min to label the ER. After three PBS washes were performed, the coverslips were mounted onto glass slides using ProLong Gold (ThermoFisher) and left to cure overnight at room temperature.

Cells were illuminated using a CoolLED pE-300 white excitation system and a Chroma 41007a HQ Cy3 filter set. An Olympus BX60, a Photometrics CoolSNAP EZ camera, a 60 × UPlanApo objective with NA = 1.4 and MetaMorph software (version 7.10.1.161) were used to image the coverslips. Exposure times of 70–100 ms were used, depending on the brightness of the cell. The pixel size of the images was 0.11 μm × 0.11 μm. Images were imported into Fiji^67^. A Gaussian filter with a 1.4 pixel radius combined with a background subtraction with a 2.4 pixel rolling ball radius were used to process the images.

### Live imaging

The videos of ER dynamics in MRC5 cells and the methods used to obtain them were described previously^2^. We also imaged ER dynamics in Vero cells, which were cultured on glass-bottomed 35 mm dishes ($\mu$-dish, No 1.5 coverslip, uncoated, Ibidi) 48 h before imaging. Cells were transiently transfected with an ER-targeted GFP construct (GFP-LongER), as described in^26^, roughly 24 h before imaging. For each dish, 400 ng of GFP-long-ER and 600 ng of a carrier DNA, pBlueScript, were combined with 4 μL of JetPEI in 100 μL of 150 mM NaCl. This solution was then incubated at room temperature for 15 min before being added to the dish.

Before imaging the dishes, pre-warmed phenol red-free imaging media was added to the dishes. This media consisted of Modified Hank’s Balanced Salt solution (with sodium bicarbonate, without phenol red, H8264, Sigma) supplemented with 10% v/v FBS, 2.5% v/v HEPES buffer (1 M, pH 7.0–7.6, H0887, Sigma), 1% MEM non-essential amino acid solution (100x, M7145, Sigma), 2% MEM amino acids (50x, M5550, Sigma), 1% Penicillin Streptomycin (10,000 units/mL, 15140122, Gibco) and 1% L-Glutamine solution (200 mM, 59202C, Sigma). An Olympus IX71 with a Cairn OptoLED light source (470 nm LED used), a Chroma ET470/40 × excitation filter and an Olympus PlanApo 100 × oil-immersion objective with a numerical aperture of 1.40 were used to view the cells. A standard green fluorescent protein filter set was placed in the emission path. An incubation chamber surrounding the microscope stage was maintained at 37 °C throughout the experiment. A Prime 95B sCMOS camera (Photometrics) was used to capture the 400–1000 frame videos with 20–30 ms exposure times and the LED continuously illuminated the sample. Metamorph (version 7.10.1.161) was used to record the 1200 × 1200 pixel videos, with a pixel size of 0.104 μm × 0.104 μm.

### Network organisation

Images were processed as described in Supplementary Note 1 and Fig. S1. The processed images were then loaded into SOAX, in which initialised contours evolve sequentially along each tubule until all tubules have been mapped. The optimal parameters were found by starting with the pre-set parameters and changing the two most important parameters^38^. These two parameters were varied to find the set that resulted in minimal tubule initialisation on background pixels and tubule contours that extended to the ends of tubules, but not beyond. If the end point of one contour was within 1 pixel of another contour, a junction was initialised. The tangent angle between the contours that met at a junction was then calculated. The minimum angle for linking snakes was set to 6 radians, so that tubules passing through junctions would not be classified as a single tubule. This matched our definition of a tubule in Fig. 1a. Two-way junctions were initialised when the tip of an evolving tubule contour overlapped with the tip of a previously identified tubule contour (described in^68^). Two-way junctions were only initialised at points of sudden changes in tubule direction. Smooth curves were not identified as junctions. Any junctions or tubules initialised on the background were removed manually.

MATLAB scripts were written to analyse the output of SOAX. The lengths of each tubule were extracted, the number of tubules converging at each junction were found and finally the angles between these tubules were calculated. MATLAB’s rangesearch function was used to find tubules starting or ending within 2 pixels of a junction. The angles between these tubules were found by performing a linear fit to the 5 points on the tubule closest to the junction and then calculating the angles between these lines. For 2-way junctions only the smallest angle was reported. Junctions consisting of 5 and 6 tubules were recorded, but these only accounted for 0.2% of the population (15 junctions out of 6756 in total).

### Point tracking

The fitting protocol was optimised as explained in Supplementary Note 2. The resulting protocol used for ER tubules was as follows. Firstly, the raw videos were imported into Fiji^67^. A Fourier transform bandpass filter with a high-pass filter of 10 pixels and a low-pass filter of 1 pixel was applied to the videos in order to highlight structures of a relevant size. These values were found to maximise the ratio of the peak to the background intensities without introducing artefacts to the images. Background subtraction using a 50 pixel rolling ball radius was then applied. This processing method improved the performance of the tracking algorithm as the signal-to-noise ratio of the images was improved (see Supplementary Note 2).

Images were then imported into MATLAB to track tubule positions. Initially, a line perpendicular to the tubule was defined by the user, along which to track the tubule position. The intensity along this line was found using bicubic interpolation, a method which takes into account the intensity in the local region of the line and resulted in a smoother intensity profile than bilinear or nearest-neighbour methods. The latter two methods often resulted in a flat section at the highest intensity, which negatively impacted the Gaussian fitting. Krig interpolation was also used to improve the fitting outcome. Raw intensity profiles across ER tubules contained very few points (usually fewer than 10) and therefore accurate fitting was challenging. To increase the number of points on the profile without altering the true values, krig interpolation was used (based on scripts from https://uk.mathworks.com/matlabcentral/fileexchange/66136-easykriging and https://uk.mathworks.com/matlabcentral/fileexchange/42885-nearestspd), resulting in a total of 100 intensity values. To ensure that the profile had a sufficiently high signal to noise ratio (SNR), the SNR of the profile was compared to a threshold found from analysing fit performance on simulated tubule profiles (see Fig. S2 and S6 in Supplementary Note 2). This threshold resulted in a fitting error of ≈1.2% (see Fig. S5). A Gaussian with an offset was fit to the intensity profile using nonlinear regression with a Cauchy weight function. This minimised fitting errors due to noise in the intensity (see Fig. S4). The position of the tubule was taken as the midpoint of the Gaussian fit. Videos of the ER in fixed cells were recorded to quantify the fitting accuracy. MSDs of tracked points were plotted and the square root of the intercept was calculated as a measure of the resolution of the tubule trajectories. The track resolution was found to be $0.04\pm 0.01$ μm (mean ± SD).

### Contour tracking

In order to track the contours of the tubules over time, a custom modification was applied to the open-source fibre tracking software, FiberApp^42^. Given two manually defined points, one at either end of a fibre, FiberApp was designed to find the spatial co-ordinates of a fibre-like object in a single image. An iterative algorithm was used to deform the contour so that it followed the image features. The modification applied to FiberApp allows the fibre to be tracked in all frames of a video and requires the user to define the start and end points of the fibre in the first frame, and then uses the tracked start and end points as an input for the next frame. The tracking parameters were optimised for videos of ER tubules in live cells (see Fig. S7 and Supplementary Note 3). The contours were saved in MATLAB for analysis. Any unexpected changes in the tubule length due to tracking errors were also minimised (see Fig. S8 and Supplementary Note 3). The results of the contour tracking were overlaid onto the original videos and each of these videos was manually checked to ensure that the contour accurately described the tubules.

In order to separate the transverse displacement from the parallel displacement, the tracked points were rotated such that the end points of the backbone lay along the x-axis. The displacements in the y-direction then corresponded to motion transverse to the tubule contour. The bias-corrected skewness, *s*, of the transverse displacement, $x$, at each tracked point, is defined as^69^

3 $$s = \frac{{\sqrt {n(n - 1)} }}{{n - 2}}\left[ {\frac{{1/n\sum\nolimits_{{i = 1}}^{n} {(x_{i} - \bar{x})^{3} } }}{{\left( {\sqrt {1/n\sum\nolimits_{{i = 1}}^{n} {(x_{i} - \bar{x})^{2} } } } \right)^{3} }}} \right],$$

where $n$ is the number of frames and $\overline{x }$ is the backbone position. To determine whether the skewness was significant, it was compared to the standard error of skewness^70^,

4 $${\sigma }_{s}=\frac{\sqrt{6n(n-1)}}{\left(n+1\right)\left(n+3\right)\left(n-2\right)}.$$

A z-score was found by taking $s/{\sigma_{s}}$, and the points were classified as skewed if the z-score was greater than 3.29 (5% significance level for larger sample sizes)^71^. The critical z-score was adjusted as ${\sigma }_{s}$ becomes very small for large datasets, so the null hypothesis of a normal distribution can easily be rejected if the standard z-score of 1.96 is used (even when the underlying distribution is not significantly different from the normal distribution).

### Fourier decomposition

The Fourier modes of each tubule were calculated using the spatial co-ordinates determined using the modified version of FiberApp. Several custom MATLAB scripts based on previous work^54,55^ were written to calculate and analyse the Fourier mode amplitudes. Firstly, the co-ordinates of the tubule at the $k$^th^ point along the backbone of the tubule, $\left({x}_{k},{y}_{k}\right)$, were used to find the tangent angle, ${\theta }_{k}$, at each point,

5 $${\theta }_{k}={\mathrm{tan}}^{-1}\left(\frac{{y}_{k+1}-{y}_{k}}{{x}_{k+1}-{x}_{k}}\right).$$

Each tubule was segmented into smaller lengths along its contour as

6 $$\Delta {s}_{k}={\left({\left({x}_{k+1}-{x}_{k}\right)}^{2}+{\left({y}_{k+1}-{y}_{k}\right)}^{2}\right)}^{1/2},$$

with the arclength at the $k$th point along the tubule, ${s}_{k}$, then defined as a summation of the segments:

7 $${s}_{k}=\sum_{j=0}^{k-1}\Delta {s}_{j}+\frac{1}{2}\Delta {s}_{k}.$$

The shape of each tubule was also expressed as a sum of Fourier modes. Cosine modes as opposed to sine modes were chosen because tubule ends are not stationary, but rather free to move with the surrounding network. An infinite sum of modes, ${\theta }_{n}\left(s\right)$, each with a mode number, $n$, and amplitude ${a}_{n}$, fully described the shape at each point of the fibre, ${\theta }_{s}$,

8 $${\theta }_{s}=\sum_{n=0}^{\infty }{\theta }_{n}\left({s}_{k}\right)=\sqrt{\frac{2}{L}} \sum_{n=0}^{\infty }{a}_{n}{\mathrm{cos}}\left(\frac{n\pi {s}_{k}}{L}\right).$$

In Eq. 8, $L$ is the length of the tubule and each mode was normalised by $\sqrt{2/{{L}}},$ such that modes were length independent. Mode amplitudes were found using the approximation that for a sufficiently large number of modes,

9 $${a}_{n}=\sqrt{\frac{2}{L}} \underset{0}{\overset{L}{\int }}ds\theta {\mathrm{cos}}\left(\frac{n\pi s}{L}\right)\approx \sqrt{\frac{2}{L}} \sum_{k=1}^{N}{\theta }_{k}\Delta {s}_{k}{\mathrm{cos}}\left(\frac{n\pi {s}_{k}}{L}\right),$$

where $N$ is the number of segments that the tubule was split into. The Nyquist-Shannon sampling theorem and the number of points tracked along the fibre limited the maximum number of modes available to model the contours. At least two points must be sampled for each mode wavelength to satisfy the first condition, such that $n\le L/\Delta s$. Gittes et al.^52^ also state that $n$ cannot be greater than $N-2$. Ten modes were used where tubules met or exceeded these conditions. For tubules too short to meet the conditions, the maximum number of modes were used that fulfilled both requirements. A script was written in MATLAB to decompose the tracked contours of the tubules into Fourier modes by finding the tangent angle, ${\theta }_{k}$, at each tracked point along the arclength of the tubule, ${s}_{k}$.

To test for sustained curvature, we examined whether the mean Fourier mode amplitudes were significantly different from zero. If $\left(\left|\langle {a}_{n}\rangle \right|-1.5*{\sigma }_{{a}_{n}}\right)>0$, where $\left|\langle {a}_{n}\rangle \right|$ is the absolute value of the mean mode amplitude and ${\sigma }_{{a}_{n}}$ is the standard deviation of ${a}_{n}$, the mode amplitude was either positive or negative for $\approx 87\%$ of frames or more. Tubules that satisfied this condition for any mode were said to have sustained curvature for that mode. Sustained curvature was most often found in the first mode, although in one case it was also found in the second mode.

### Itemset mining

Association rules state that if some property, A, is true for a tubule, some other property, B, is also likely to be true. A and B are referred to as the antecedent and consequent respectively. The significance of these rules is quantified using support, confidence and lift. Support is defined as the number of times a set of items occurs divided by the total number of transactions (in this case, tubules). Confidence is a measure of how often a rule applies and is defined as the number of transactions containing both A and B, divided by the number of transactions containing A. Finally, lift compares the probability of A and B occurring independently to the observed frequency of the combination. Mathematically, lift is defined as the ratio of the support for the whole itemset (A and B) to the product of the support for the A and the support for B. A value greater than 1 indicates that the variables occur together more often than expected.

To find association rules for ER tubules, a matrix was created in which columns referred to the tubule properties and each row represented a single tubule. A value of 1 indicated that the tubule had the property whereas 0 meant that this property was not detected for the given tubule. The matrix contained 8 columns, relating to the presence (or lack of) the four properties defined in the text. Association rules were found using scripts from https://uk.mathworks.com/matlabcentral/fileexchange/42541-association-rules with some additions. Some of the properties measured only occurred in very few tubules, so the minimum support required for a rule was set to 0.1 such that rules would still be generated even if they only applied to very few tubules. The minimum confidence however was set to 0.9 to ensure that this did not result in rules that only applied rarely.

## Supplementary Information

Supplementary Information.

## Data Availability

Software for point tracking, contour tracking and Fourier analysis of filaments along with videos of the ER in live cells are available in a GitHub repository at https://github.com/htperkins/tubule-tracker. Further videos analysed during this study are available upon request from the corresponding author.
