# Supplementary material for: Network organisation and the dynamics of tubules in the endoplasmic reticulum
Source: Sci Rep. 2021 Aug 10;11:16230. doi: 10.1038/s41598-021-94901-2 (PMC8355327; doi:10.1038/s41598-021-94901-2)
Supplement: Supplementary file 1 — Supplementary Information. [file 41598_2021_94901_MOESM1_ESM.pdf]

Supplementary Information: Network Organisation and the Dynamics of Tubules in the Endoplasmic Reticulum

Hannah T. Perkins[1,2], Victoria J. Allan[2]*, Thomas A. Waigh[1]*

[1] Biological Physics, Department of Physics and Astronomy, Schuster Building, The University of Manchester, Oxford Road, Manchester, M13 9PL

[2] Division of Molecular and Cellular Function, School of Biological Sciences, Michael Smith Building, The University of Manchester, Dover Street, Manchester, M13 9PT

*Corresponding authors: viki.allan@manchester.ac.uk and t.a.waigh@manchester.ac.uk

Supplementary Note 1: Image Processing for Tubule Segregation

Before using SOAX to trace the tubules and junctions of the network, images were processed such that the nucleus and thicker regions of the network were excluded (see Figure S1). A mask containing the brightest pixels of the image (usually the denser network surrounding the nucleus) was created and these pixels were removed from the images. Firstly, to select these higher intensity regions, Otsu thresholding was used. Holes in the resulting mask were filled using ImageJ's Fill Holes function. Small dots in the thresholded image were removed by subtracting particles with an area of less than 1000 $pixels^2$ from the mask. To increase the signal to noise ratio of tubules in the original image, a Fourier bandpass filter with a high threshold of 15 pixels and a low threshold of 3 pixels was used. The background was then subtracted using a 50-pixel rolling ball algorithm. The low threshold of the Fourier bandpass filter was set slightly higher for these images than for tubule tracking as very thin tubules were not initialised as well in SOAX. The pixels within the mask were then removed from the processed image, resulting in only the tubular region of the network remaining. Without these processing steps, tubules were often initialised on the out-of-plane tubules in the nuclear region and thicker sections of the network, although no clear tubules could be seen by eye.

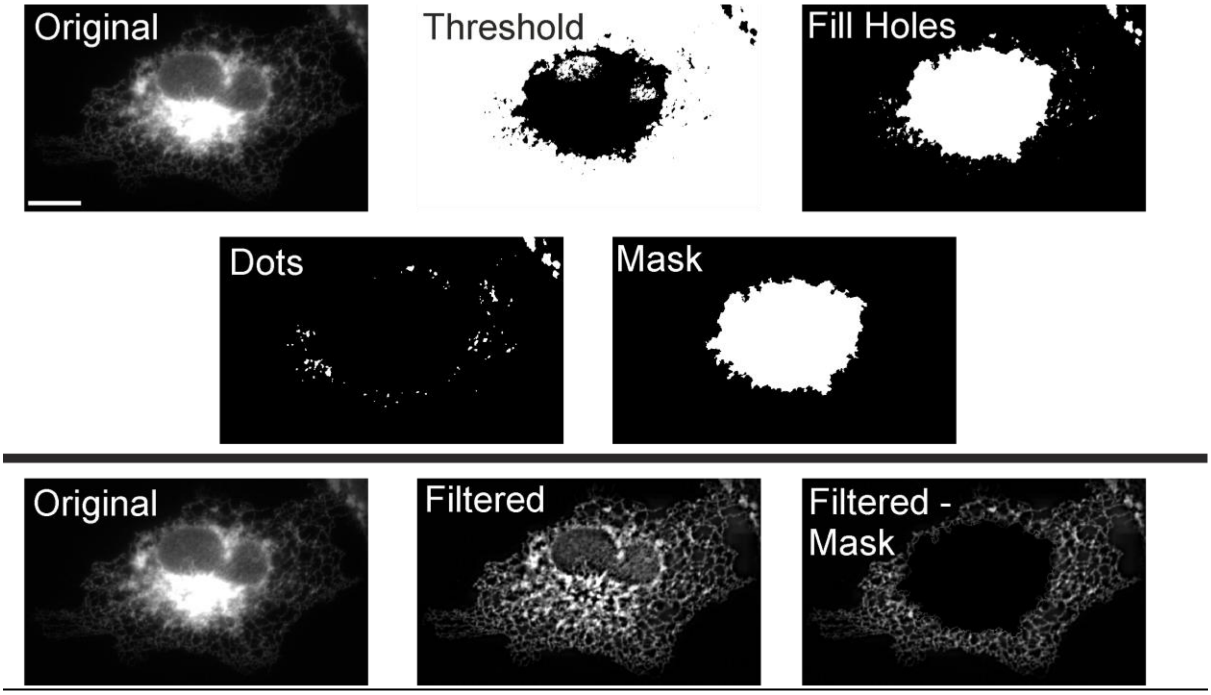

Figure S1. Pre-processing of images for SOAX. The top panel shows method for selecting the nucleus and thicker regions of the ER network. The bottom panel shows how this mask was used on the filtered original image. A Fourier bandpass filter (high threshold of 15 pixels, low threshold of 3 pixels) and 50 pixel rolling ball background subtraction were applied to the original image, resulting in the filtered image. Scale bar 10 µm.

Supplementary Note 2: Intensity Profile Fitting

To optimise the tracking of ER tubules in one dimension, intensity profiles across tubules were simulated and different fitting protocols were tested. The protocol that minimised the error in the fitted centre of a Gaussian was used. Initially, several simple Gaussian profiles with the same centre points, but varying peak and edge values were simulated. It was found that the profiles with the highest peak-to-background ratio, or signal to noise ratio (SNR), had the smallest difference between the true and fitted centre points. To find the relationship between the SNR and the error in fitting and to compare fitting methods, 20 Gaussians with SNR values increasing from 1 to 20 were simulated (see Fig. S2). The number of points along the profile was chosen such that the simulated profiles had a similar sampling density to intensity profiles taken from experimental data. Random noise with an amplitude of 10% of the peak value was added to replicate the noise seen in experiments. These 20 Gaussian profiles were then copied and random noise was added to the edge values to simulate edge effects (or kickbacks) seen in experiments, usually due to other parts of the network being detected at the edges of the line. The added noise was positive with an amplitude of 25% of the maximum intensity.

A Gaussian with an offset was used to model the simulated Gaussians. No background removal techniques were used at this stage as they were found to erode points from the tubule profile as well as from the noisier edges. This resulted in fewer points on the intensity profile to fit and therefore an increase in the fitting error. An offset was added to the model as without background removal, the intensity at the profile edges was nonzero. The parameters of the fit were initialised as following (see Fig. S3): the offset was set as the minimum intensity value, the mean of the Gaussian

was set as the position along the line at which the maximum intensity occurred and the amplitude was set as the difference between the maximum and minimum intensity. The width of the Gaussian

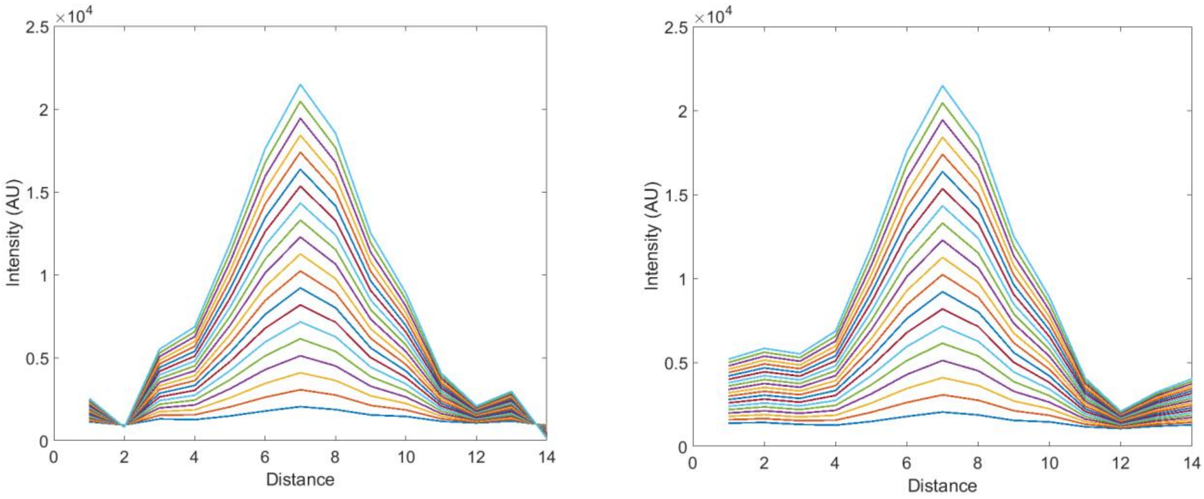

Figure S2. Example of simulated Gaussians with random noise and signal to noise ratios from 1 to 20. Edge effects are added to the Gaussians in the image on the right to simulate noise due to other parts of the network entering the region of the profile.

was estimated by finding where the profile first crossed half of the amplitude, searching from the maximum intensity outwards in both directions. The difference in distance between these points was then halved to find the initial guess for the width. The fit converged successfully much more often with the parameters initialised in this way. The width initialisation was particularly crucial as noise at the edges occasionally influenced width estimations using other methods. MATLAB's nonlinear fitting function was used with these parameter initialisations. Within this function, there is an option to use robust fitting, in which outliers are given less weight in the fitting procedure. Eight robust weight functions were tested on the simulated data as well as non-robust fitting. The Cauchy weight function improved the fit accuracy over non-robust fitting for both the datasets with and without edge effects (see Fig. S4), although the difference was minimal for the latter. This was the only robust weight function for which the fit accuracy was improved for both datasets and for which accuracy increased monotonically with an improvement in SNR. Therefore, a Gaussian function with an offset was used with Cauchy robust nonlinear fitting to track points on tubules.

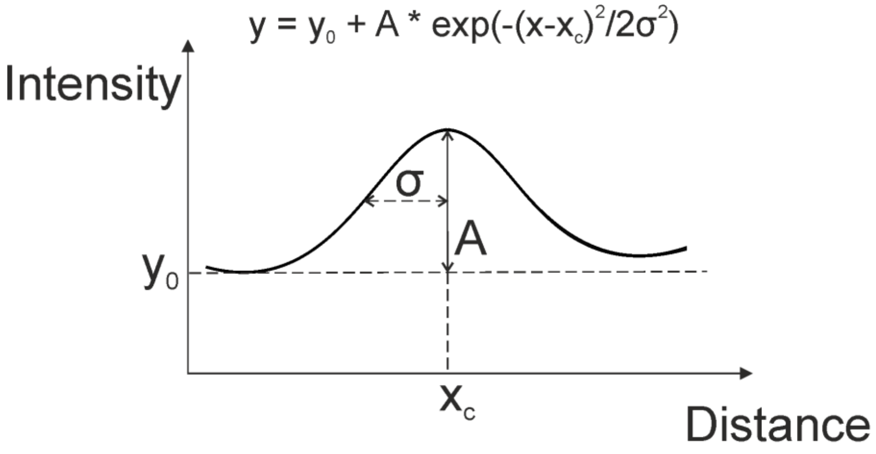

Figure S3. Model used to fit ER tubule profiles and initial parameter guesses shown on an example profile.

The increase in fitting accuracy for the simulated data was minimal after the SNR (the ratio of the peak to background intensity before noise was added) exceeded 5 (see Fig. S5). For a SNR of 5 in the simulated data, the error in the estimation of the centre point was roughly 1.2%. We decided to set the minimum SNR for real data to 5. However, the SNR cannot be directly measured for real data as edge effects make the background intensity difficult to define. Instead, we used integrated intensity as a measure of SNR in real data. To calculate the integrated intensity, the most prominent minima on either side of the peak were found. If there were no minima, the edge values were used. A line joining these points was found and the area between this line and the intensity profile was calculated. This integrated intensity scaled linearly with the SNR for the simulated data (see Fig. S6). The threshold SNR for analysis of ER tubules was set slightly above the maximum integrated intensity value for SNR=4, to ensure that the real data would have an SNR of at least 5. The distribution of ER tubule widths is narrow[1] and therefore the difference in peak and background intensities was the most influential factor in the integrated intensity of experimental data.

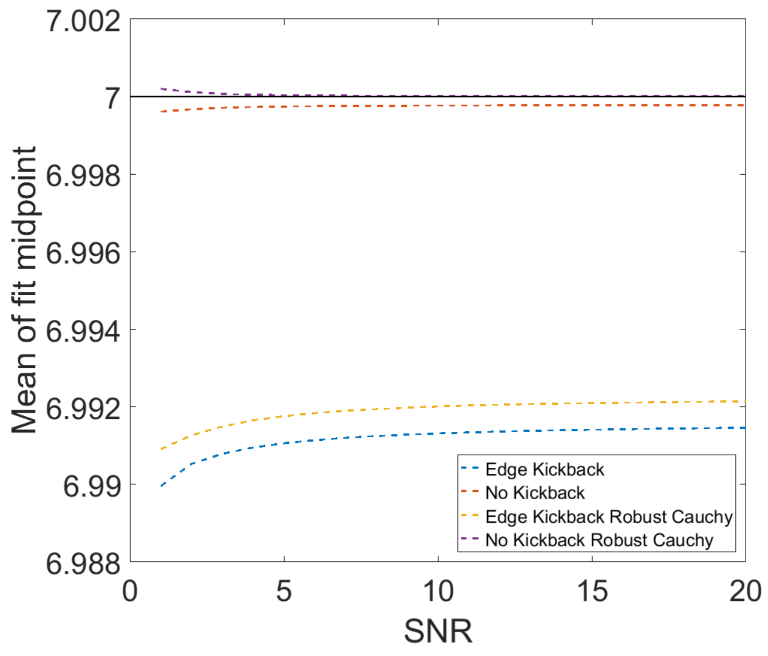

Figure S4. Fitting accuracy comparison for simulated profiles using non-robust nonlinear fitting or robust fitting with a Cauchy weight function. The true value of the midpoint was 7. The mean fit midpoints were found from 5000 repeats at each SNR value.

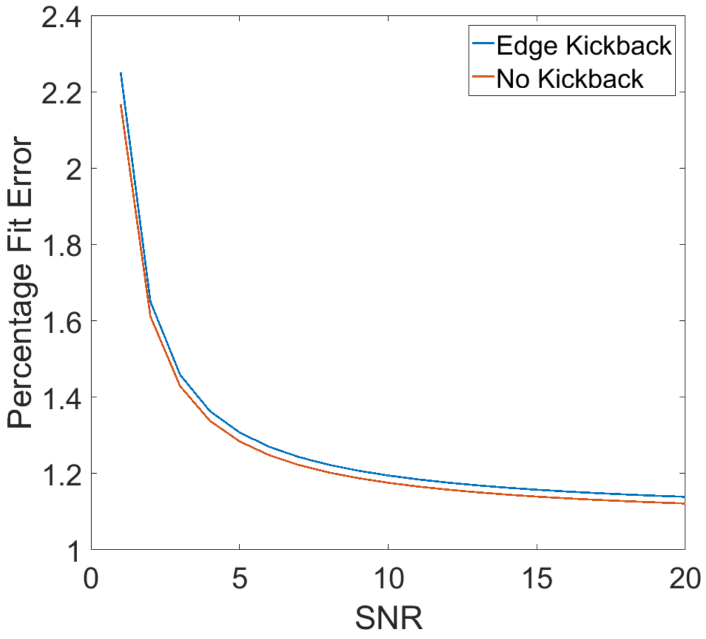

Figure S5. Percentage error in midpoint fit for different SNR values. The mean percentage fit error of 1000 repeats was used for each SNR value.

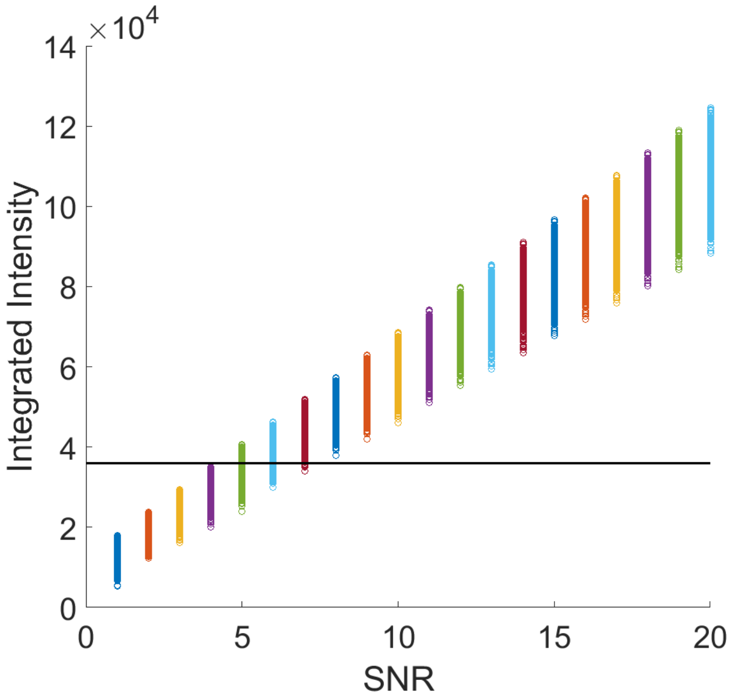

Figure S6. Calculated integrated intensity against simulated signal to noise ratio. The values scale linearly, with some variation in the integrated intensity due to the noise added to each profile. The black line shows the threshold used for real data. Only data with an integrated intensity above this threshold was analysed.

Supplementary Note 3: Optimisation of Contour Tracking

The fitting parameters defined in FiberApp[2] were optimised for fitting ER tubules by performing a parameter scan. A video of a single tubule was used for the parameter testing. The ground truth

contour for the final frame of the video was manually defined and then compared to the tracked contour for each parameter set. Two metrics were used to measure the success of the tracking. The first was simply the distance between the tracked ends of the tubule and the true ends of the tubule. The distance for the start point and the final point were added to give a measure of the erroneous shrinking or elongating of the tubule. The second was the Frechet distance, a measure of similarity between contours[3,4]. Initially, a coarse-grained scan was performed, involving a large range for each of the parameters. Both resulting metrics were normalised for the population of coarse-grained results so that neither was more strongly weighted, as the magnitude of the total distance was occasionally much larger than that of the Frechet distance. The normalised values were summed for each parameter set and two sets were found to perform best. A fine-grained scan based on these results was then performed using the same method. This resulted in four parameter sets performing almost equally well. Finally, these parameter sets were tested in more detail by looking at the tracking over all frames of the video (see Fig. S7). A parameter set was chosen that most accurately followed the contour without developing spurious harsh bends or excessive smoothing over the contour.

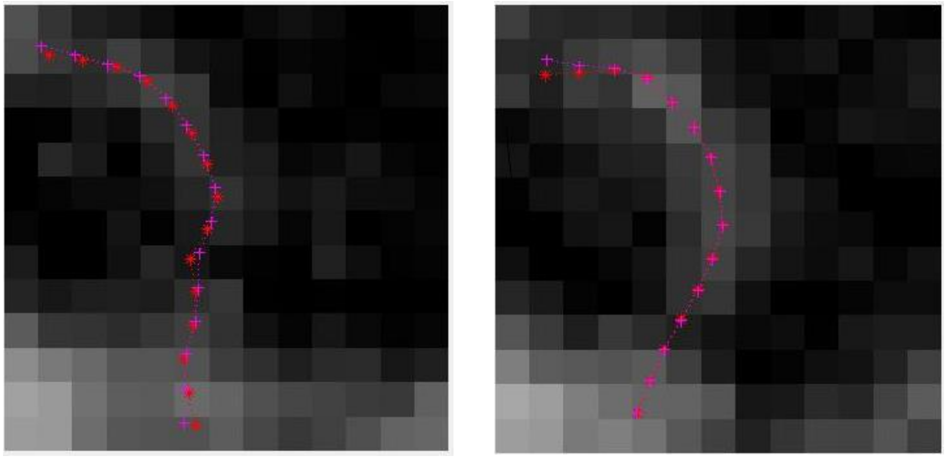

Figure S7. Tracking results of two different parameter sets in two frames of a video. The red track has a harsh bend in the frame on the left and an excessive downward tilt in the frame on the right. The pink track more accurately follows the tubule contour.

In this work, established tubules were chosen for analysis, so the contour length was not expected to change significantly. However, due to the end points of the tubule being user-defined for the first frame, occasionally the length of the tubule would change significantly in the first few frames before settling to a contour. To exclude these artificial length changes from the analysis, if the number of points tracked changed length by more than one point during the first 5% of frames, the tracked frames before the change were discarded. This excluded any transient errors due to tubule initialisation. Occasionally, the tubule length also changed by one point in later frames. These slight variations are to be expected due to unavoidable low levels of noise in the videos. To minimise these tracking errors parallel to the tubule contour, the Frechet distance was used to find the tracked positions that best matched the tubule position before the length change. For example, if the length of the tubule changed from N to N+1 points, the Frechet distance between the N points in the first frame and points 1 to N in the second frame would be compared to the Frechet distance between the N points in the first frame and points 2 to N+1 in the second (see Fig. S8). The pair that minimised the Frechet distance would be the most similar and only these points used would be kept.

In the example in Fig. S8, the points in the 21st row would be removed. Together, these two methods minimised tracking errors parallel to the tubule contours.

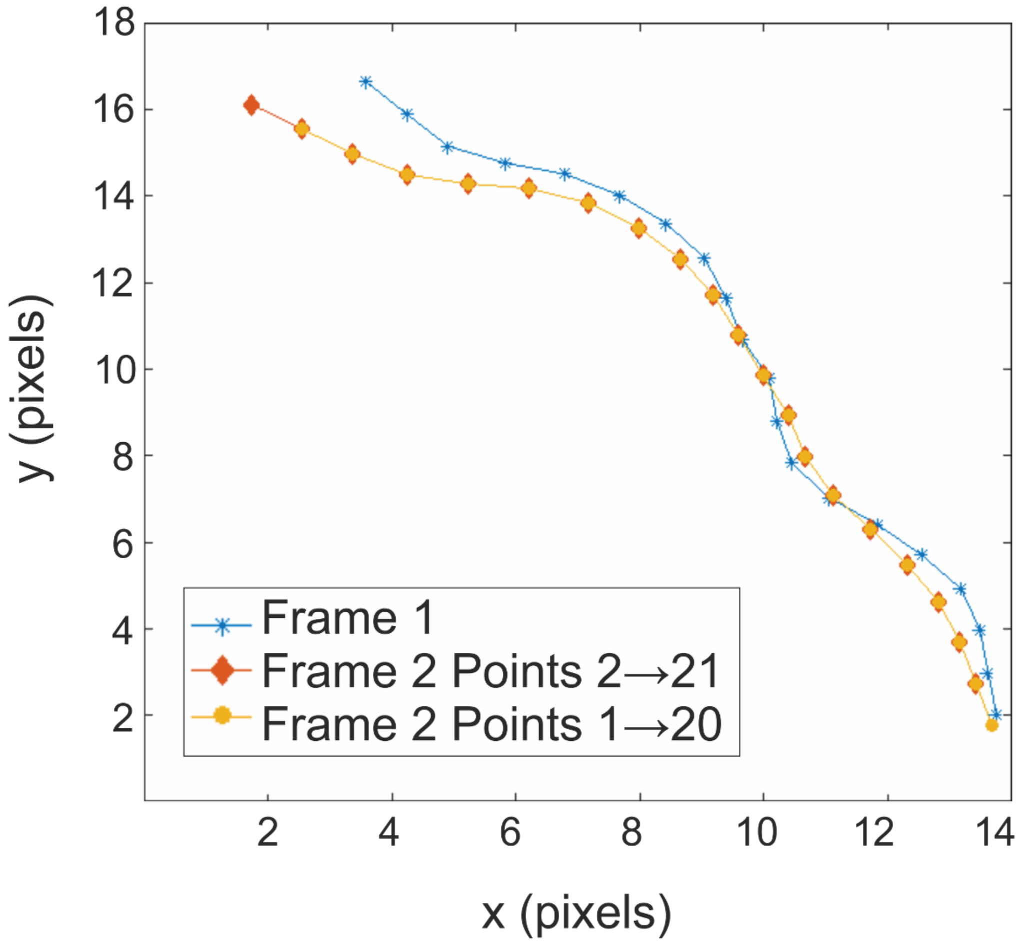

Figure S8. Illustration of the protocol used when changes in length were detected. The tubule elongated from 20 to 21 points between frame 1 and frame 2. The contour from the first frame was compared with the first 20 points and the last 20 points from the second frame. In this case, the first 20 were most similar to the contour in the first frame.

<u>References</u>

1. Georgiades, P. *et al.* The flexibility and dynamics of the tubules in the endoplasmic reticulum. *Sci. Rep.* **7**, 16474 (2017).
2. Usov, I. & Mezzenga, R. FiberApp: An open-source software for tracking and analyzing polymers, filaments, biomacromolecules, and fibrous objects. *Macromolecules* **48**, 1269–1280 (2015).
3. Mosig, A. & Clausen, M. Approximately matching polygonal curves with respect to the Fréchet distance. *Comput. Geom. Theory Appl.* **30**, 113–127 (2005).
4. Alt, H. & Godau, M. Measuring the resemblance of polygonal curves. *Eighth Annu. Symp. Comput. Geom.* 102–109 (1992). doi:10.1145/142675.142699
